# ISG15 promotes tumor progression via IL6/JAK2/STAT3 signaling pathway in ccRCC

**DOI:** 10.1007/s10238-024-01414-z

**Published:** 2024-06-29

**Authors:** Wei Xie, Yuanfeng Zhang, Zhechuan Zhang, Qinke Li, Lesha Tao, Ronggui Zhang

**Affiliations:** 1 Department of Urology, The Second Affiliated Hospital of Chongqing Medical University, Linjiang Road. 74, Jiangbei, Chongqing, China; 2 Department of Urology, Chongqing People’s Hospital, Xingguang Road.118, Chongqing, China; 3 Department of Gynaecology and Obstetrics, The Second Affiliated Hospital of Chongqing Medical University, Linjiang Road. 74, Yuzhong, Chongqing, China

**Keywords:** ISG15, Clear cell renal cell carcinoma, IL6/JAK2/STAT3, Proliferation, Migration, Invasion

## Abstract

Although renal cell carcinoma (RCC) is a prevalent type of cancer, the most common pathological subtype, clear cell renal cell carcinoma (ccRCC), still has poorly understood molecular mechanisms of progression. Moreover, interferon-stimulated gene 15 (ISG15) is associated with various types of cancer; however, its biological role in ccRCC remains unclear.This study aimed to explore the role of ISG15 in ccRCC progression.ISG15 expression was upregulated in ccRCC and associated with poor prognosis. RNA sequence analysis and subsequent experiments indicated that ISG15 modulated IL6/JAK2/STAT3 signaling to promote ccRCC proliferation, migration, and invasion. Additionally, our animal experiments confirmed that sustained ISG15 knockdown reduced tumor growth rate in nude mice and promoted cell apoptosis. ISG15 modulates the IL6/JAK2/STAT3 pathway, making it a potential therapeutic target and prognostic biomarker for ccRCC.

**Supplementary Information:**

The online version contains supplementary material available at 10.1007/s10238-024-01414-z.

## Introduction

Renal cell carcinoma (RCC) is a prevalent malignant neoplasm in the human population [1]; the pathological subtype clear cell renal cell carcinoma (ccRCC) accounts for the largest proportion [2]. Although surgical removal or ablation can effectively treat early-stage ccRCC with a positive outlook, some patients may still experience disease recurrence or distant metastasis after surgery [3]. Furthermore, the development of drug resistance and metastatic spread often lead to fatal outcomes in the majority of cases [4]. To identify novel therapeutic targets for patients with ccRCC, it is imperative to investigate the fundamental mechanisms and discover potential diagnostic and prognostic biomarkers.

Interferon-stimulated gene 15 (ISG15), a 15 kDa protein, is essential for post-translational modification of various proteins [5]. Additionally, when not bound, ISG15 promotes cytokine production and enhances immune cell activation [6]. Numerous studies have elucidated the cancer-causing function of ISG15 in different types of tumors, like breast cancer [7], esophageal squamous cell carcinoma [8], hepatocellular carcinoma [9], nasopharyngeal carcinoma [10], pancreatic cancer [11], and ovarian tumor [12]. In the future, ISG15 may be used as a molecular target for treating ccRCC; however, a deeper understanding of its mechanisms is necessary.

The STATs (signal transducer and activator of transcription) family includes STAT1, STAT2, STAT3, STAT4, STAT5A, STAT5B and STAT6 [13, 14]. Several studies have demonstrated that STAT1 and STAT2 maintain an effective and long-lasting immune response and antitumor defense [15–20]. Conversely, STAT3 and STAT5 have been implicated in a positive correlation with the initiation and progression of various solid tumors, particularly STAT3 [13]. Various studies have demonstrated STAT3’s important role in the prognosis and progression of human tumors such as gastric [21], lung [22], colorectal [23], ovarian [24], cervical [25], and hepatocellular carcinoma [26]. The Janus kinases (JAKs) family, consisting of JAK1, JAK2, TYK2 and JAK3, are involved in regulating STAT activation [13]. The JAK2/STAT3 pathway has been recognized as a key player in the progression of different types of solid tumors in humans.

In this study, ISG15 showed increased expression in ccRCC tissues and cell lines. Excessive expression was found to indicate a poor outlook in individuals with ccRCC. Furthermore, blocking ISG15 function suppressed the migration, proliferation, and invasion of ccRCC cells and increased cell death. Moreover, we found that the biological changes in ccRCC after inhibiting ISG15 are mainly a result of regulating JAK2/STAT3 signaling. Additionally, we demonstrated that silencing of ISG15 could impede the growth of ccRCC tumors in animal models. Therefore, ISG15 may become a new therapeutic target for ccRCC in the future.

## Material and methods

### Clinical samples

Immunohistochemistry (IHC) was performed on 20 ccRCC samples from different Tumor Node Metastasis (TNM) stages provided by the Pathology Department of the Second Affiliated Hospital of Chongqing Medical University (CQMU). The specimens obtained from the patients were verified to have ccRCC at initial diagnosis, and none of them had received chemotherapy or radiotherapy. Moreover, the frozen tissues’ IHC results were appraised by two pathologists based on the staining intensity and percentage of positive cells. Ethical approval was obtained from the Medical Ethics Committee of the Second Affiliated Hospital of the CQMU.

### Bioinformatics and RNA-sequence (RNA-seq) analysis

RNA-seq expression profiles and clinical data were downloaded from the Cancer Genome Atlas (TCGA) database (https://portal.gdc.cancer.gov/) for 537 kidneys renal clear cell carcinoma (KIRC) and 72 adjacent non-tumor tissues. ISG15 expression in KIRC was analyzed using R software (version 3.6.3) based on the downloaded data in HTSeq-fragment per kilobase per million format from the TCGA database. Survival analysis was performed based on RNA-seq and survival data downloaded from TCGA.

Differentially expressed genes for Kyoto Encyclopedia of Genes and Genomes (KEGG) analyses were obtained from stable ISG15-knockdown OSRC2 cell RNA-seq data.

### Cell culture

Five ccRCC cell lines (CAKi-1, A498, 786O, 769P and OSRC2) and one normal renal tubular epithelial cell line (HK2) were cultured. A cell culture medium composed of Gibco Roswell Park Memorial Institute (RPMI)-1640 medium, 10% fetal bovine serum and 1% penicillin–streptomycin solution was used. For further analysis, cells were cultured at 37 °C in 5% CO_2_.

OS-RC-2 and 769-p cell lines were provided by Pricell. The cell lines were authenticated using GeneMapper IDX software and compared with the ATCC, DSMZ, JCRB, and Cellosaurus databases.

### Transfection in vitro

ISG15-shRNA and lentiviruses, produced by Hanheng Biologics, were used to knock down ISG15 in OSRC2 cells with the following target sequences (5′ → 3′): #1 CTGAGCATCCTGGTGAGGAAT, #2 CATGTCGGTGTCAGAGCTGAA, #3 GTGGTGGACAAATGCGACGAA, and vector group TTCTCCGAACGTGTCACGTAA. The cells were placed in a dish for cultivation, and the medium was replaced once the cell concentration reached 30–50%. We calculated the virus volume using the MOI value before adding it to OSRC2 cells, which were then incubated for 4 h. The remaining medium was added after the initial incubation period. After incubation for an additional 24 h, the medium was changed, and puromycin was added for screening. After 48 h of infection, an OSRC2 cell line with stable ISG15 knockdown was obtained by altering the medium.

A lentivirus overexpressing ISG15 was engineered and produced by Qingke Bio. Following infection with the virus using the previously described steps, puromycin was introduced for screening purposes 48 h post-infection, and the cell culture medium was replaced 96 h post-infection to establish a stable 769-P cell line overexpressing ISG15.

To confirm the knockdown and overexpression efficiency, real-time quantitative polymerase chain reaction (RT-qPCR) and western blot analysis were performed after transfection.

### Western blotting

Approximately 20–40 µg of overall cellular protein was isolated and separated by electrophoresis on a 10% polyacrylamide gel prior to transfer onto a polyvinylidene fluoride (PVDF) membrane for western blot examination. After blocking with a quick blocking solution, the PVDF membrane was incubated overnight with the primary antibody at 4 °C on a shaker. After washing the PVDF membrane with Tris-buffered saline containing Tween, we incubated it for 1 h with secondary antibodies and used CLINX ChemiScope S6 to visualize the protein bands. The purchasing information for antibodies is as follows: Anti-β-Actin (Cat#60,008-1-Ig, Proteintech), Anti-ISG15 (Cat#285,367, Abcam), Anti-ZEB1 (Cat#203,829, Abcam), Anti-N-cadherin (Cat#22,018-1-AP, Proteintech), Anti-Vimentin (Cat#60,330-1-Ig, Proteintech), Anti-Snail (Cat#13,099-1-AP, Proteintech), Anti-E-cadherin (Cat#20,874-1-AP, Proteintech), Anti-Slug (Cat#9585 T, CST), Anti-Twist2 (Cat#11,752–1-AP, Proteintech), Anti-JAK2 (Cat#3230, CST), Anti-STAT3 (Cat#9139, CST), Anti-Bcl2 (Cat#23,309, Zenbio), Anti-Bax(Cat#380,709, Zenbio), Anti-Caspase3/p19 (Cat#19,677-1-AP, Proteintech), Anti-p-JAK2 (Cat#32,101, Abcam), Anti-p-STAT3 (Cat#267,373, Abcam), and Anti-IL-6 (Cat#290,735, Abcam).

### 5-Ethynyl-2′-deoxyuridine (EdU) and cell counting kit-8 (CCK-8) proliferation assays

The ability of ccRCC cells to multiply was evaluated using the CCK-8 and EdU proliferation tests. After transfection, ccRCC cells were seeded in 96-well dishes and incubated for 24–72 h. After reaching 24 h multiples (0, 1, 2, and 3), cells in the 96-well plate were treated for 2 h with APExBIO’s CCK-8 reagent at 37 °C. The absorbance at 450 nm was measured using a Thermo Fisher LUX Multimode Microplate Reader. The EdU proliferation test was performed to determine the proliferation capacity of ccRCC cells. Cells were seeded in 24-well plates and grown until they filled the wells. EdU assays were conducted using RIBOBIO’s Cell-Light EdU Apollo In Vitro Kit. Images were taken using a Nikon inverted fluorescence microscope.

### Migration and invasion assays

Initially, wound healing assays were performed to determine the migratory capacity of stable silenced-ISG15 and overexpressed-ISG15 ccRCC cells. After transfection, the 6-well dishes were used for seeding cells, which were then cultured until they reached 90–100% confluency. Afterward, the monolayer was scraped with a 200 μL pipette tip, and the basic RPMI-1640 medium was added following a rinse with phosphate-buffered saline (PBS). Images of cell scratches were captured using an inverted microscope at 0 and 24 h.

We further evaluated the migration and invasion of stable ISG15-knockdown and stable ISG15-overexpression ccRCC cells using a transwell assay. For the migration test, 500 μL of complete medium was placed in each well of 24-well dishes, and Transwell chambers with 200 μL of basic medium and 4000 ccRCC cells were inserted into the dishes. After culturing at 37 °C for 12 h, the transwell chambers were fixed with 4% paraformaldehyde for 20 min and stained with 0.5% crystal violet for 30 min. Residual cells in the chambers were wiped with cotton buds, and images were captured using an inverted microscope. For the invasion assay, Matrigel was placed in the transwell chambers, and the same procedure was performed as in the migration assay.

### Quantitative polymerase chain reaction in real time(RT-qPCR)

RNA was isolated from ccRCC cells using Agbio’s SteadyPure Quick RNA Extraction Kit. After cDNA synthesis using Takara’s PrimeScript® RT reagent kit, the cDNA products were amplified by a two-step PCR method using Bio-Rad’s CFX Connect and Takara’s SYBR GreenTM Premix Ex TaqTM II. β-actin served as an intra-group control in the experiment. The primers used were as follows: β-actin, forward (F) 5′-TGT GGC ATC CAC GAA ACT AC-3′, reverse (R) 5′-GGA GCA ATG ATC TTG ATC TTC A-3′; ISG15, F, 5’-CGC AGA TCA CCC AGA AGA TCG-3′ and R, 5′-TTC GTC GCA TTT GTC CAC CA-3′.

### Flow cytometry

During the cell cycle, the cells were seeded in 6-well plates and collected in Eppendorf tubes when they reached full growth. Samples were resuspended in 100 μL of pre-chilled PBS per tube, 900 μL of 75% pre-chilled ethanol was added to each tube for cell fixation, and the samples were then sent to the flow cytometry laboratory for cycle testing. For apoptosis, cells collected in Eppendorf tubes were resuspended in 500 μL of pre-chilled PBS and then sent to the low cytometry laboratory for apoptosis testing.

### Animal experiments

The animal experiments were approved by the Laboratory Animal Management and Use Committee of the Second Affiliated Hospital of CQMU. Eight nude mice were randomly assigned to two groups: vector and shISG15. Ten million tumor cells were suspended in 100 μL of PBS and injected into the skin of the mice. We monitored the mice weekly for tumor formation and measured their long and short diameters to determine the tumor volume. After four observation cycles, mice were euthanized, tumors were collected and weighed, and IHC and TUNEL assays were performed on tumor tissues.

### IHC and TUNEL assays

Using the EnVision kit (Dako), IHC staining was performed using Anti-ISG15 (Cat#15,981-1-AP, Proteintech) and anti-proliferating cell nuclear antigen (PCNA) (Cat#10,205-2-AP, Proteintech). For the three-step peroxidase procedure, hematoxylin was used as a counterstain and adhered to the manufacturer’s instructions. The ImageJ software was used to determine the expression levels of PCNA and ISG15 based on the staining intensity.

For the terminal deoxynucleotidyl transferase dUTP nick-end labeling (TUNEL) assay (Cat#C1098, Beyotime), the amount of apoptosis in xenograft tumor frozen sections was determined following the manufacturer’s instructions.

### Statistical analysis

In this study, experimental tests were conducted thrice, and the findings are reported as average ± standard deviation unless specified otherwise. GraphPad Prism (version 8) was used to analyze the data and determine the significance level for all experiments using the Student’s t-test. A significance level of *p* < 0.05 was used for all experiments, with statistical significance denoted as follows: ns (statistically non-significant, (*p* > 0.05), * (*p* < 0.05), ** (*p* < 0.01) and *** (*p* < 0.001)).

## Results

### ISG15 upregulation predicted poor prognosis in ccRCC

**Fig. 1:**
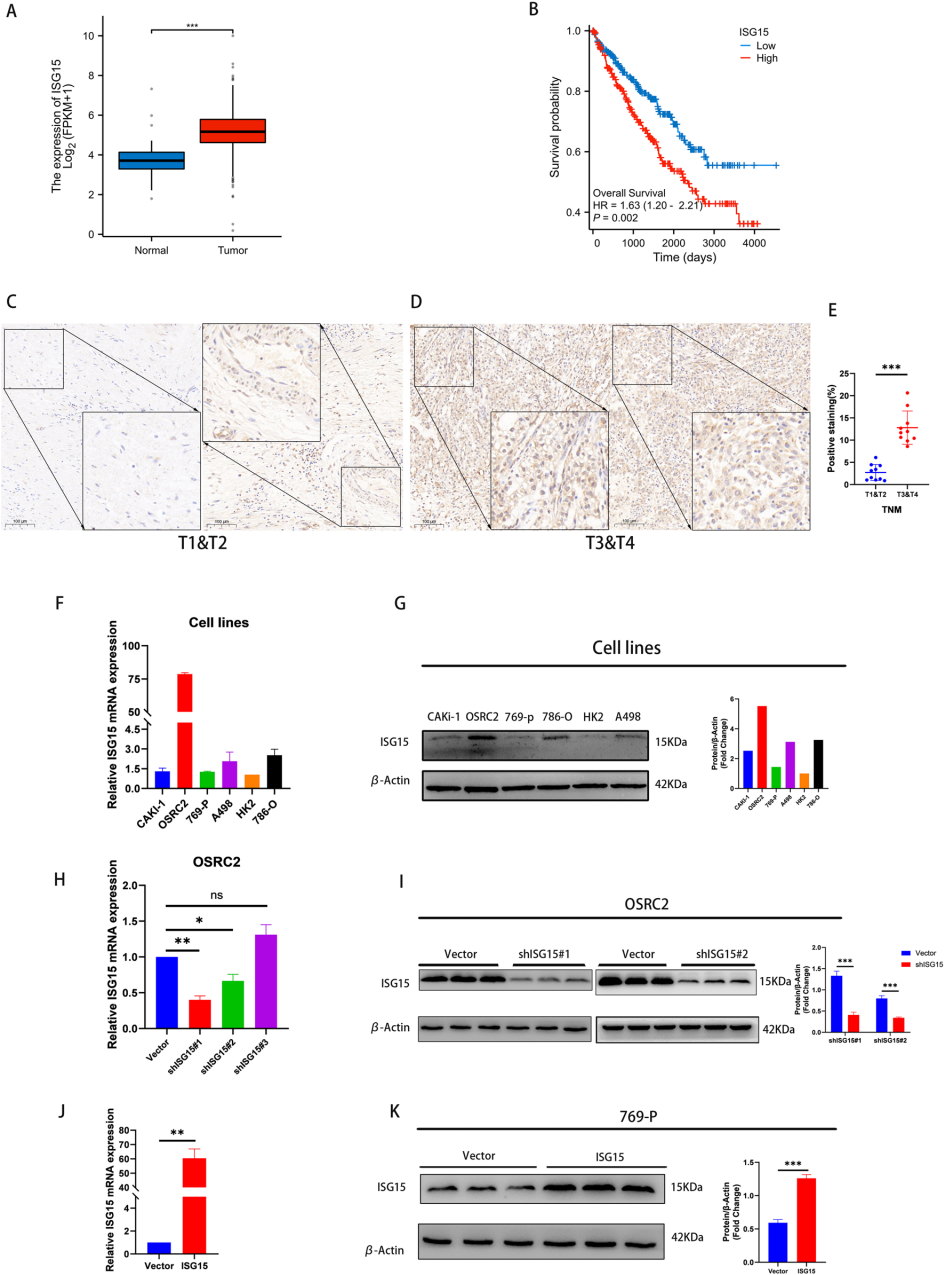
ISG15 level is increased in ccRCC and associated with poor prognosis. The expression of ISG15 among tumors and corresponding normal tissues in the TCGA database (**A**) and upregulated ISG15 predicts poor prognosis (**B**). (**C–E**) The expression of ISG15 was lower in the T1 and T2 stages than in the T3 and T4 stages in patients with ccRCC. The expression of ISG15 in ccRCC cells was verified by RT-qPCR (**F**) and western blotting analyses (**G**). (**H–K**) OSRC2 and 769-P cells were chosen to construct stable transgenic cell models, and the efficiency was identified by RT-qPCR (**H** and **J**) and western blot analyses (**I** and **K**) (*p < 0.05, **p < 0.01, ***p < 0.001)

Bioinformatics analysis showed a significant increase in ISG15 levels in ccRCC tissues compared to normal tissues (Fig. 1A). Additionally, a negative relationship was observed between ISG15 levels and patient outcomes in ccRCC (Fig. 1B). After immunohistochemical staining in our group, it was found that ISG15 levels increased steadily as the stage of patients with ccRCC advanced (Fig. 1C–E). These results suggested that increased ISG15 levels in ccRCC could be used as a predictor of unfavorable prognosis.

### ISG15 promoted the ccRCC proliferation

RT-qPCR and western blotting were used to evaluate the expression of ISG15 in five human ccRCC cell lines (OSRC2, 786-O, A498, 769-P, and Caki-1) and a normal renal epithelial cell line (HK2). Among the ccRCC cell lines, OSRC2 and 769-P showed the highest and lowest ISG15 expression levels, respectively, as indicated by the results in Fig. 1F, G. Stable ISG15-knockdown cell lines were created using the OSRC2 cell line, while stable ISG15-overexpression cell lines were generated using the 769-P cell line (Fig. 1H–K).

**Fig. 2:**
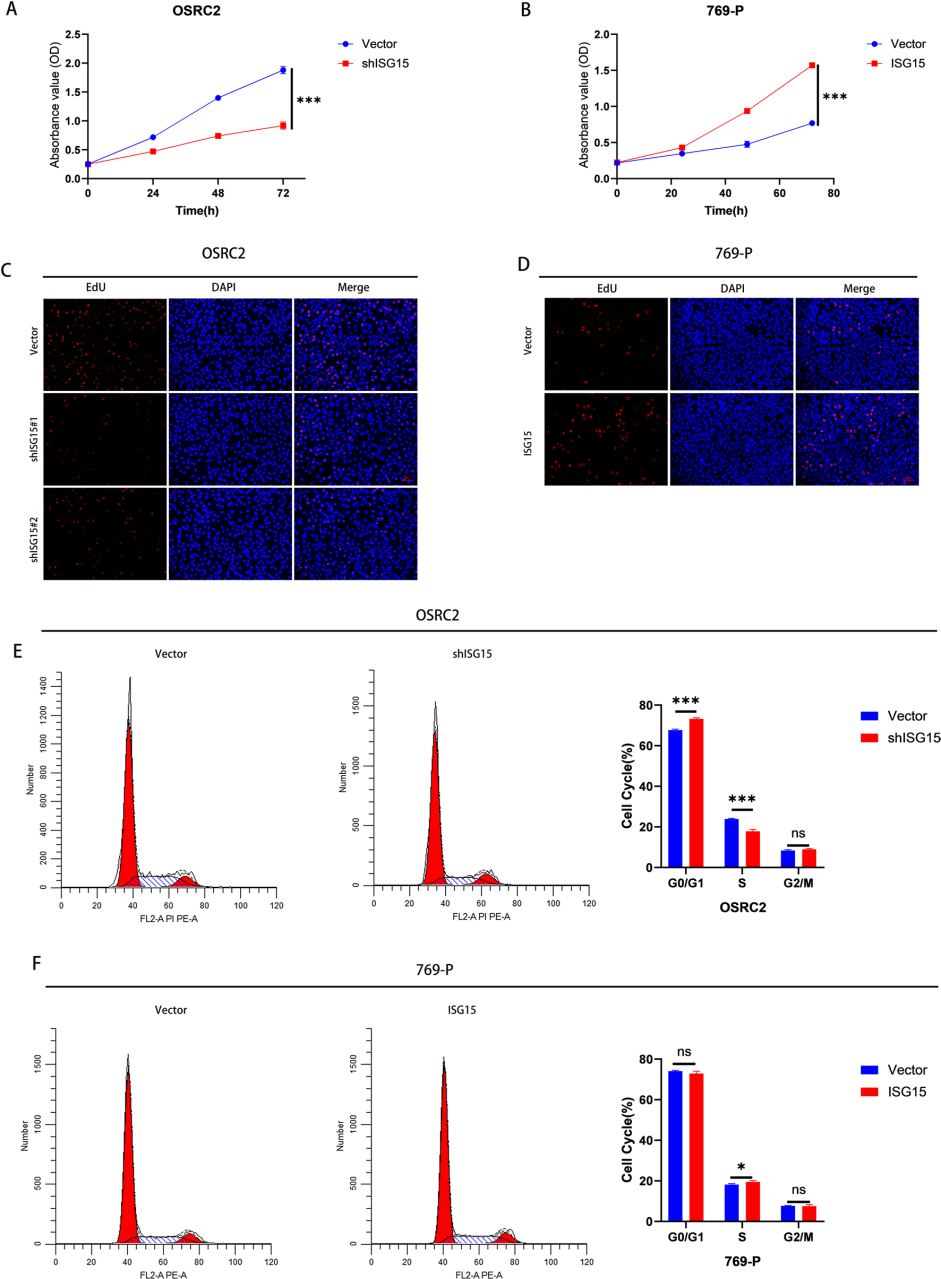
ISG15 promotes the proliferation of ccRCC. (**A–D**) CCK8 and EdU assays for stable ISG15 knockdown OSRC2 cells (**A** and **C**) and stable ISG15 overexpressed 769-P cells (**B** and **D**). Flow cytometry assays of the cell cycle of stable ISG15 knockdown OSRC2 cells (**E**) and stable ISG15 overexpressed 769-P cells (**F**). *p < 0.05, **p < 0.01, ***p < 0.001

To understand ISG15’s biological role in ccRCC proliferation, we examined the proliferation capacity of stable ISG15-knockdown and ISG15-overexpression ccRCC cells using the CCK-8 and EdU proliferation assays. ISG15 downregulation hindered the proliferation of ccRCC cells, whereas its upregulation enhanced their proliferation (Fig. 2A–D). Subsequent evaluation of the cell cycle in ccRCC cells with silenced and overexpressed ISG15 using flow cytometry further elucidated the positive impact of ISG15 on the cell proliferation capacity (Fig. 2E, F). The results suggested a positive correlation between ISG15 levels and ccRCC cell growth.

### ISG15 inhibited the ccRCC cell death

**Fig. 3:**
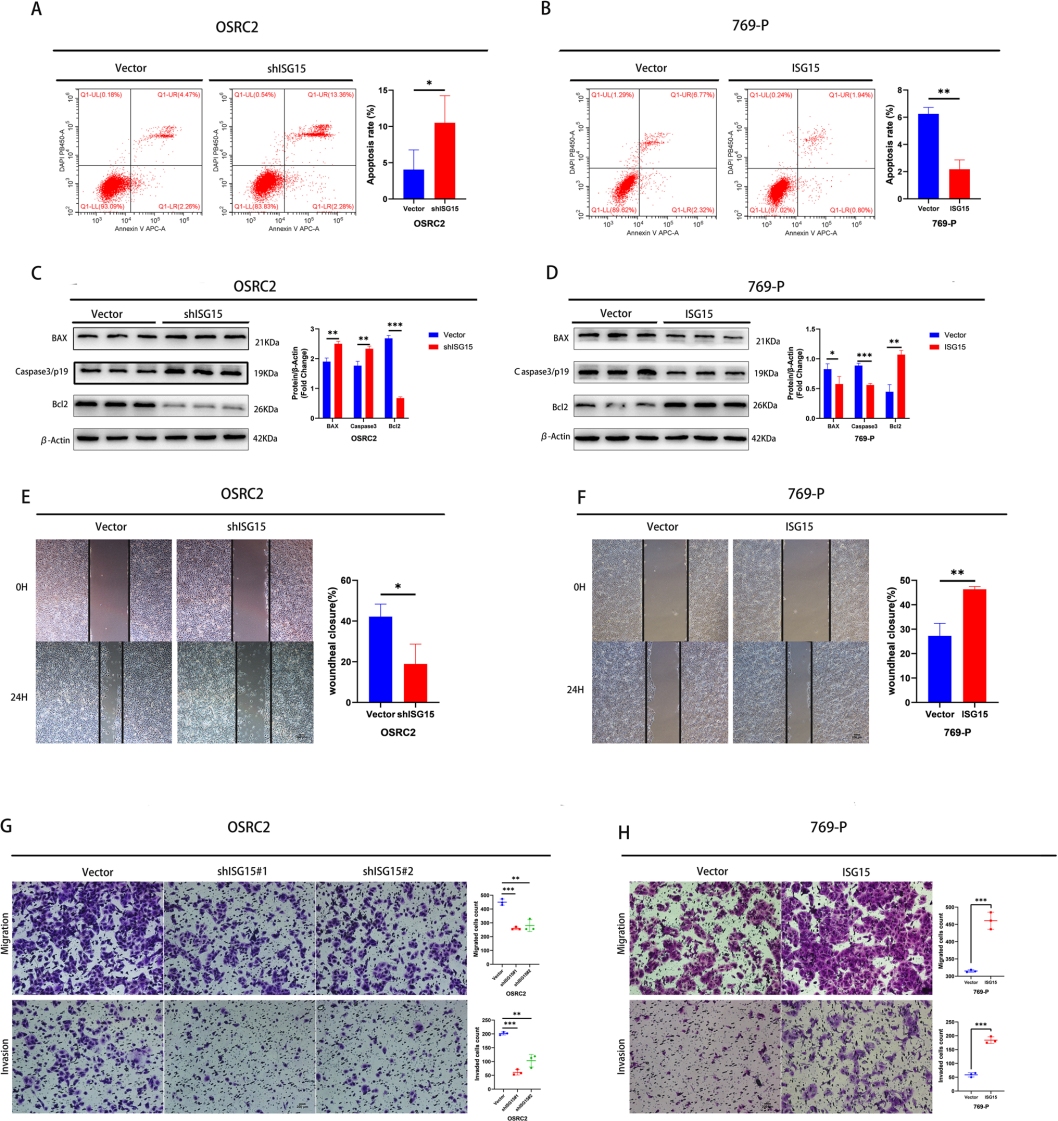
ISG15 promotes the migration and invasion of ccRCC cells but inhibits their apoptosis**.** Flow cytometry assays for apoptosis of stable ISG15 knockdown OSRC2 cells (**A**) and stable ISG15 overexpressed 769-P cells (**B**). (**C–D**) Western blotting for Bax, Bcl2, and caspase-3/p19. (**E** and **F**) Wound healing assays in stable ISG15 knockdown OSRC2 cells (**E**) and stable ISG15 overexpressed 769-P cells (**F**). (**G** and **H**) Transwell assays for stable ISG15 knockdown OSRC2 cells (**G**) and stable ISG15 overexpressed 769-P cells (**H**). Scale bar = 100 μm. *p < 0.05, **p < 0.01, ***p < 0.001

We further investigated the biological role of ISG15 in programmed cell death in ccRCC cells. Flow cytometry was used to evaluate apoptotic cell death in ccRCC cells exhibiting both silenced and upregulated ISG15 expression, indicating a negative association between ISG15 and programmed cell death in ccRCC (Fig. 3A, B). Moreover, western blot analysis was used to measure the expression of pro-apoptotic proteins Caspase-3/p19, Bax, and the anti-apoptotic protein Bcl-2 in silenced-ISG15 and overexpressed-ISG15 cells. The findings indicated that suppressing ISG15 led to a reduction in Bcl-2 levels, along with an elevation in Bax and caspase-3/p19 expression (Fig. 3C). Contrarily, these effects were reversed by ISG15 overexpression (Fig. 3D). The results provided valuable insight into the role of ISG15 in ccRCC programmed cell death.

### ISG15 promoted ccRCC invasion and migration

To further understand the biological role of ISG15 in ccRCC, transwell and wound healing assays were conducted to evaluate the migration and invasive abilities of stable ISG15-knockdown and stable ISG15-overexpression ccRCC cells. We found that ISG15 silencing suppressed ccRCC cell migration and invasion (Fig. 3E, G). However, ISG15 overexpression promoted migration and invasion of ccRCC cells compared to ISG15 knockdown (Fig. 3F, H). These results confirmed that ISG15 is an essential factor in promoting ccRCC cell migration and invasion.

### Correlation between ISG15 and epithelial-to-mesenchymal transition (EMT) in ccRCC

**Fig. 4:**
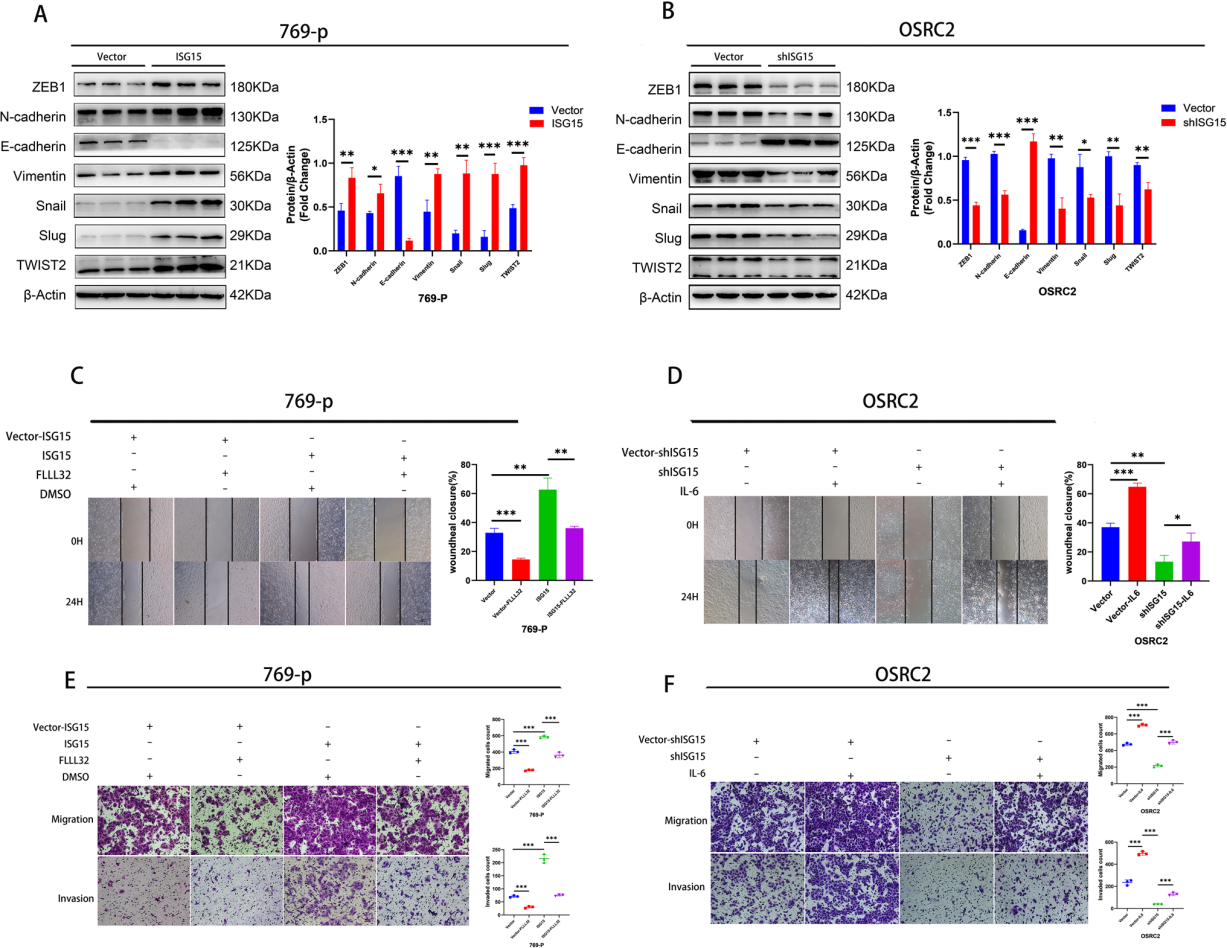
ISG15 promotes the migration and invasion of ccRCC via the IL6/JAK2/STAT3 signaling pathway and may be involved in EMT. (**A–B**) Western blotting assays for EMT markers ZEB1, N-cadherin, E-cadherin, Vimentin, Snail, Slug, and TWIST2. (**D**and **F**) ccRCC cells with stable ISG15 knockdown were treated with recombinant human IL-6 (10 ng/mL) for 24 h and harvested for subsequent studies. Wound healing (**D**) and Transwell assays (**F**) for cell migration and invasion. (**C** and **E**) ccRCC cells with stable ISG15 overexpression were treated with FLLL32 (1 μM) for 48 h and then harvested for subsequent studies. Wound healing (**C**) and Transwell assays (**E**) for cell migration and invasion. *p < 0.05, **p < 0.01, ***p < 0.001

Numerous studies have shown the notable influence of EMT on ccRCC progression. We used western blotting analysis to identify different EMT biomarkers and to investigate the functional role of ISG15 in EMT in ccRCC. Western blotting analysis showed that ISG15 overexpression increased the levels of mesenchymal biomarkers (N-cadherin, Zeb1, Vimentin, Snail, Slug, and Twist2) and decreased E-cadherin levels (an epithelial biomarker) (Fig. 4A). In contrast, decreased ISG15 levels resulted in different outcomes (Fig. 4B). Our findings suggested that ISG15 may have a significant impact on EMT in ccRCC.

### ISG15 may promote the ccRCC progression via IL6/JAK2/STAT3 signaling pathway

**Fig. 5:**
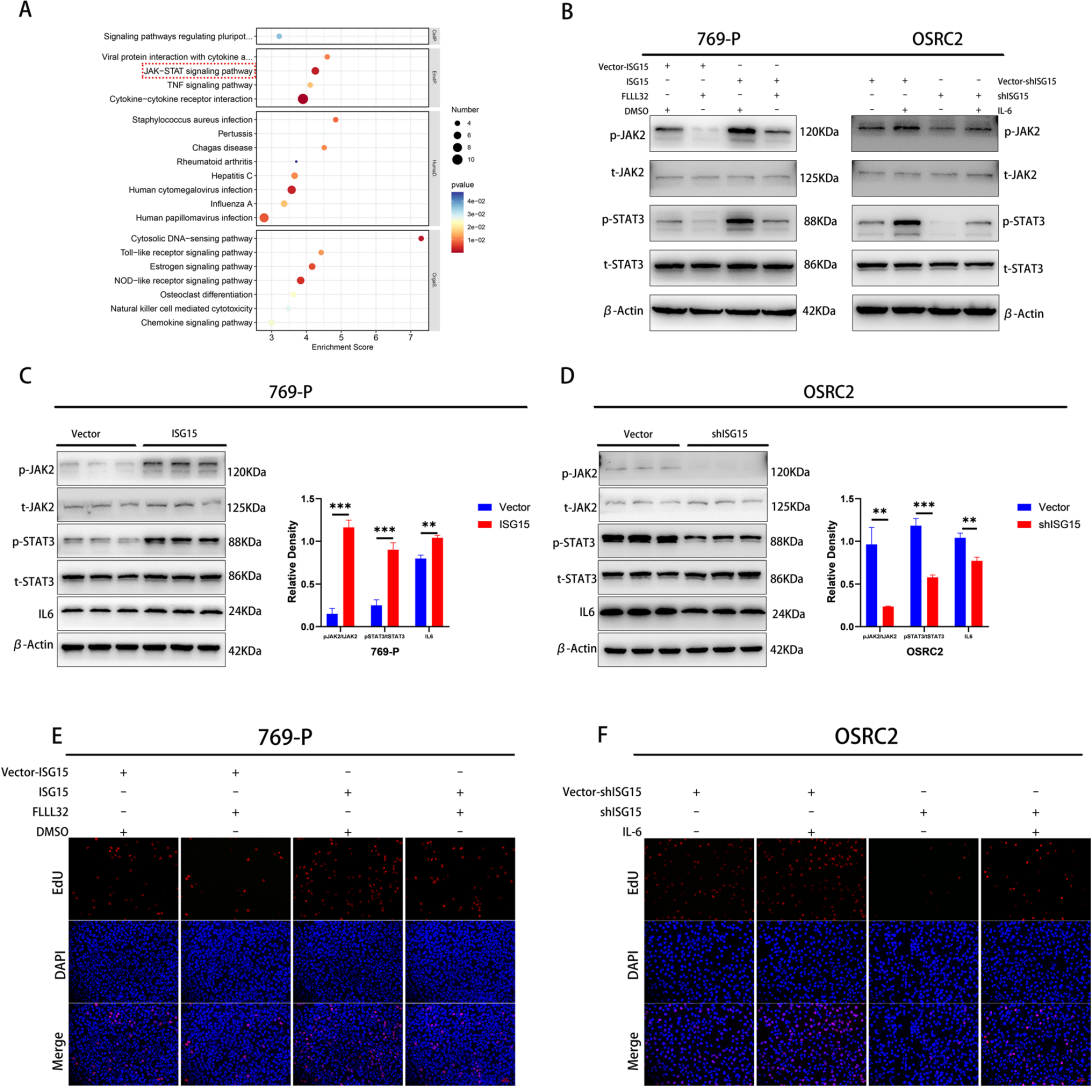
ISG15 may be involved in IL6/JAK2/STAT3 signaling pathway in ccRCC and promotes its proliferation. (**A**) The top 20 signaling pathways with significant differences were shown in KEGG. (**C** and **D**) Western blotting for IL6, JAK2, STAT3, p-JAK2, and p-STAT3. ccRCC cells with stable ISG15 overexpression were treated with FLLL32 (1 μM) for 48 h, and ccRCC cells with stable ISG15 knockdown were treated with recombinant human IL-6 (10 ng/mL) for 24 h and then harvested for further studies. (**B**) Western blotting for JAK2, STAT3, p-JAK2, and p-STAT3. (**E,F**) EdU assays for cell proliferation. *p < 0.05, **p < 0.01, ***p < 0.001

To determine the specific mechanisms by which ISG15 promotes ccRCC progression, RNA-seq and bioinformatic analyses were conducted. According to the findings shown in Fig. 5A, ISG15 appears to play a role in the JAK/STAT signaling pathway in ccRCC. After integrating the results of RNA-seq analysis with existing studies, the differentially expressed genes interleukin 6 (IL-6), JAK2, and STAT3, which were downregulated by ISG15 knockdown, were identified for further analysis. Moreover, western blotting results revealed that when ISG15 was silenced, IL6 expression, p-JAK2, and p-STAT3 proteins were reduced. However, total JAK2 (t-JAK2) and total STAT3 (t-STAT3) levels remained unchanged (Fig. 5D). Elevated ISG15 levels increased IL6, p-JAK2, and p-STAT3 proteins. In contrast, t-JAK2 and t-STAT3 levels remained unchanged (Fig. 5C). These findings suggested a potential role in ccRCC for ISG15 in regulating IL6/JAK2/STAT3 signaling.

To determine whether ISG15 contributes to the progression of ccRCC by regulating the JAK2/STAT3 signaling pathways, we used the JAK2/STAT3 pathway inhibitor FLLL32 and the activator recombinant human IL6 in rescue experiments. In EdU, wound healing, and transwell assays, ISG15 overexpression promoted proliferation, migration, and invasion of ccRCC cells; however, this promotion was inhibited by adding FLLL32 (Figs. 4C, E and 5E). Furthermore, IL-6 successfully restored the decreased migration, invasion, and proliferation of ccRCC cells induced by the ISG15 knockdown (Figs. 4D, F, and 5F). Additionally, JAK2 and STAT3 phosphorylated forms showed similar alterations, with no changes observed in the protein levels of t-JAK2 and t-STAT3 (Fig. 5B), which were identified using western blotting. In conclusion, ISG15 might enhance ccRCC progression through IL6/JAK2/STAT3 signaling.

### ISG15 promoted tumor growth in vivo

**Fig. 6:**
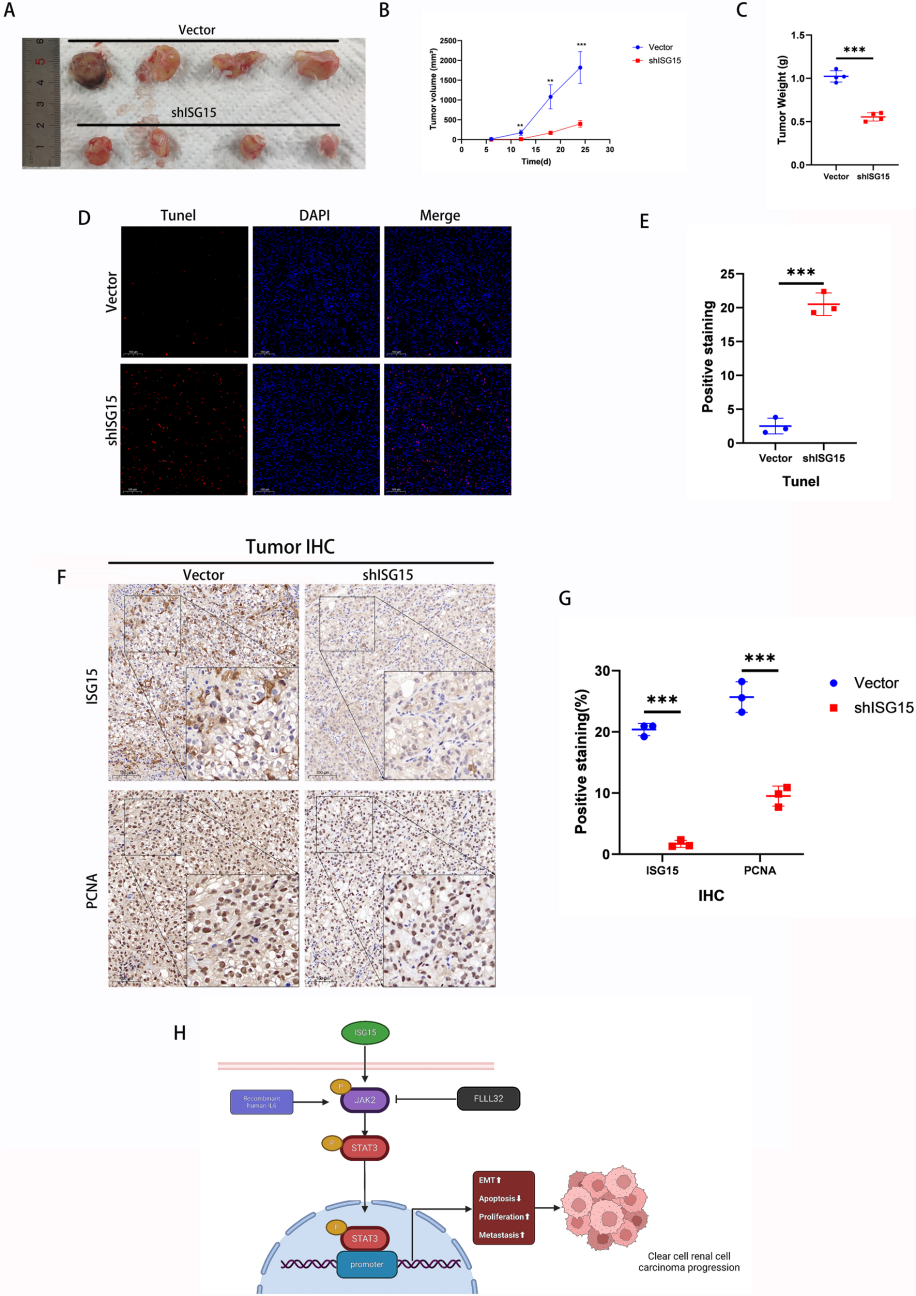
ISG15 promotes ccRCC tumor growth and inhibits cell death in vivo. (**A**) Images of tumor tissues. (**B**) Tumor growth rates were measured. (**C**) Tumor weight recording. (**D–G**) TUNEL (**D–E**), IHC staining for ISG15, and PCNA (**F–G**) in tumor sections. (**H**) The proposed working model indicates the oncogenic function of ISG15 in ccRCC by regulating the IL6/JAK2/STAT3 signaling pathway. *p < 0.05, **p < 0.01, ***p < 0.001

By injecting vector and shISG15 OSRC2 cells under the skin of immunodeficient nude mice, we investigated the possible role of ISG15 in promoting the proliferation of ccRCC in vivo. We found that mice in the vector group had faster tumor growth than mice in the shISG15 group (Fig. 6A, B). Immunohistochemical analysis of the tumors was performed after euthanizing the mice, and the tumors were weighed (Fig. 6C). Subsequent findings revealed that ISG15 inhibited the induction of apoptosis in ccRCC (Fig. 6D, E) while demonstrating a stimulatory effect on ccRCC cell proliferation (Fig. 6F, G). In accordance with these results, it was suggested that ISG15 could facilitate cell proliferation and impede programmed cell death in ccRCC in vivo.

## Discussion

Upon initial diagnosis, approximately 20–30% of individuals with RCC have advanced stages of the disease due to its gradual development and vague symptoms [27]. Despite the initial success of treatment, recurrence and metastasis significantly complicate the treatment of RCC, resulting in poor prognosis [28]. Incomplete comprehension of the metastatic process of RCC poses a significant obstacle to the effective treatment of this condition. The primary difficulty in treating ccRCC is the suppression of metastasis. Identifying a promising therapeutic molecular target is essential for improving patient outcomes and treatment effectiveness through early intervention, prognostication, and metastasis suppression in ccRCC.

The gene ISG15 encodes a 15 KDa-sized protein that participates in post-translational modifications of various proteins and enhances its stability through binding to related proteins. Several studies have shown that ISG15 plays a critical role in the development and progression of multiple cancers; however, how it regulates ccRCC is unknown. Hence, we conducted a comprehensive investigation of the biological functions of ISG15 in ccRCC. Our findings indicated that ISG15 functions as an oncogene in ccRCC and may hold promise as a therapeutic target for this disease in the future. Furthermore, using bioinformatics analysis, we observed elevated ISG15 expression levels in ccRCC, which exhibited a strong association with unfavorable patient prognosis. Using immunohistochemical staining, we analyzed ISG15 expression in ccRCC clinical specimens. Our study revealed a positive correlation between ISG15 expression and ccRCC progression. RT-qPCR and western blot analyses were used to confirm ISG15 expression in the five ccRCC cell lines. Based on these results, we selected OSRC2 and 769-P cell lines for further investigation of the biological functions and specific mechanisms of ISG15 in ccRCC.

In recent studies, ISG15 has been demonstrated to play a significant role in the regulation of cell proliferation and metastasis in various human cancers. Furthermore, the biological activity of ISG15 has been found to vary across different cancer types [29–34]. Moreover, recent studies indicate that ISG15 may serve as a potential prognostic factor for adverse outcomes in individuals with ccRCC [35, 36]. Consequently, it is crucial to investigate the precise biological role of ISG15 in ccRCC. To address this issue, we hypothesized that ISG15 exerts a stimulatory influence on cancer-related traits like cell proliferation, invasion, and metastasis in ccRCC. Subsequently, we conducted a comprehensive analysis of ISG15’s involvement in ccRCC by employing various methodologies, including bioinformatics analysis, RNA-seq, and in vitro and in vivo experiments. In our investigation, we observed that ISG15 downregulation resulted in a notable suppression of proliferation, invasion, and migration in ccRCC cells while promoting apoptosis.

Conversely, ISG15 overexpression led to reverse phenotypic alterations in these cells. Subsequently, a ccRCC cell line with stable ISG15 knockdown was used to establish a nude mouse xenograft model. The tumor volume and growth rate were measured and compared between the shISG15 and vector groups. Tumors were collected for weight measurement, IHC analysis, and TUNEL apoptosis detection. The results indicated a significantly slower growth rate, lower volume, and lower weight, as well as decreased ISG15 and PCNA staining in the shISG15 group compared to the vector group. Furthermore, the TUNEL apoptosis assay indicated a significantly higher apoptosis rate in the knockdown group than in the control group. These results substantiate the hypothesis that ISG15 is vital for proliferation, invasion, migration, inhibition of apoptosis, and tumor growth in ccRCC. Additionally, KEGG enrichment analysis of the RNA-seq results revealed a strong association between ISG15 and the JAK/STAT signaling pathway.

The study of how cells react to interferon has led to the discovery of the JAK/STAT pathway and has shown a cell signaling paradigm that extends from bacteria to humans. Several JAK and STAT signals linked to human malignancies have been identified in recent years through in-depth research of the JAK/STAT signaling system [37–40]. Among these, JAK2 and STAT3 have been widely studied in human solid tumors [41–47]. The transcription factor STAT3, which is involved in controlling the proliferation, migration, and invasion of various human malignancies, is activated by the protein tyrosine kinase JAK2. Consequently, we investigated whether the impact of ISG15 on tumorigenesis and metastasis is mediated by targeting the JAK2/STAT3 signaling pathway. Initially, western blot analysis was used to verify the influence of changes in ISG15 expression levels on the abundance of proteins associated with the JAK2/STAT3 pathway. Using western blotting, we found that down- or upregulation of ISG15 altered p-JAK2 and p-STAT3 protein levels, while t-JAK2 and t-STAT3 protein levels remained the same. We subsequently employed the JAK2/STAT3 signaling pathway inhibitor FLLL32 in a rescue experiment within the ISG15 overexpression group to further confirm whether ISG15’s impact on the proliferation, invasion, and migration capabilities of ccRCC is mediated through the regulation of the JAK2/STAT3 signaling pathway. The findings of our study indicated that the increased proliferation, migration, and invasion of ccRCC cells resulting from ISG15 overexpression is notably suppressed by FLLL32. Furthermore, various research studies have demonstrated the role of IL-6 in activating the JAK2/STAT3 signaling pathway [41, 42, 45, 46, 48]. Hence, recombinant human IL-6 was used in a rescue experiment within the ISG15 knockdown group. Recombinant IL-6 could rescue the inhibitory effects of ISG15 knockdown on ccRCC proliferation, migration, and invasion. Accordingly, ISG15 may promote the proliferation and metastasis of ccRCC by regulating the IL6/JAK2/STAT3 signaling pathway.

The current research findings indicated that ISG15 is crucial for tumors [11, 49]. To investigate whether ISG15 is involved in programmed cell death and cell cycle regulation in ccRCC, we performed flow cytometry analysis of the knockdown ISG15 and overexpression ISG15 group, respectively. Moreover, we used western blot analysis to assess the changes in the expression levels of Bcl2 and Bax, as well as the activated form of caspase-3, namely caspase-3/p19. Furthermore, ISG15 knockdown inhibited cell cycle progression and activated apoptosis in ccRCC cells, reducing the anti-apoptotic protein Bcl2 and enhancing the pro-apoptotic proteins Bax and caspase-3/p19. However, ISG15 elevation led to an opposite result, suggesting that ISG15 participates in regulating apoptosis and cell cycle progression in ccRCC cells.

Furthermore, multiple studies have shown that STAT3 activation can inhibit apoptosis in various human malignancies [50–52] and that STAT3 activation is also involved in the regulation of the cell cycle[39, 53], suggesting that STAT3 plays an indispensable role in maintaining tumor survival and promoting tumor progression. Therefore, we hypothesized that ISG15 inhibits ccRCC apoptosis and promotes cell cycle progression by regulating the activation of STAT3, thereby promoting ccRCC proliferation. Thus, ISG15 may play an essential role in ccRCC apoptosis and cell cycle progression.

In conclusion, ISG15 was upregulated in ccRCC, correlating with adverse survival outcomes and prognostic implications in patients with ccRCC. Furthermore, we demonstrated that ISG15 controls proliferation, migration, invasion, apoptosis, and cell cycle progression in ccRCC via modulating the IL-6/JAK2/STAT3 signaling pathway (Fig. 6H). Therefore, personalized treatment strategies targeting ISG15 are expected to improve the efficacy of ccRCC treatment. However, this study still has some limitations. First, we demonstrated that ISG15 induced ccRCC progression via the IL-6/JAK2/STAT3 pathway; however, the precise mechanism by which activated STAT3 affects ccRCC phenotypic changes is unknown. Furthermore, although in vivo experiments were conducted using a nude mouse xenograft model, ISG15’s role in the metastasis of ccRCC remains unexplored. Moreover, more experiments are needed to determine the mechanism in vivo by which ISG15 regulates ccRCC progression whether it is the same as in vitro*.*

## Conclusions

Our research confirmed the role of ISG15 as a cancer-causing gene in ccRCC and conducted a comprehensive analysis to identify its specific mechanisms. This study demonstrated that ISG15 regulates IL6/JAK2/STAT3 signaling to promote the proliferation, migration, and invasion of ccRCC cells. In conclusion, ISG15 might be a promising therapeutic target for ccRCC.

## Supplementary Information

Below is the link to the electronic supplementary material.

Supplementary file 1 (DOCX 19 kb)

Supplementary file 1 (DOCX 19 kb)

Supplementary file 1 (DOCX 19 kb)

## Data Availability

The data of this study are available from the corresponding author upon reasonable request.
