# Supplementary material for: ISG15 promotes tumor progression via IL6/JAK2/STAT3 signaling pathway in ccRCC
Source: Clin Exp Med. 2024 Jun 29;24(1):140. doi: 10.1007/s10238-024-01414-z (PMC11217101; doi:10.1007/s10238-024-01414-z)
Supplement: Supplementary file 3 [file 10238_2024_1414_MOESM3_ESM.docx]

Woundhealing.zip
